# Inadequate birth spacing is perceived as riskier than all family planning methods, except sterilization and abortion, in a qualitative study among urban Nigerians

**DOI:** 10.1186/s12905-017-0439-2

**Published:** 2017-09-11

**Authors:** Hilary M. Schwandt, Joanna Skinner, Luciana Estelle Hebert, Lisa Cobb, Abdulmumin Saad, Mojisola Odeku

**Affiliations:** 1 Center for Communication Programs, Johns Hopkins Bloomberg School of Public Health, Baltimore, Maryland USA; 2 Fairhaven College, Western Washington University, 516 High Street, MS 9118, Bellingham, WA 98225 USA; 3 Center for Interdisciplinary Inquiry and Innovation in Sexual and Reproductive Health, Section of Family Planning and Contraceptive Research, Department of Obstetrics & Gynecology, University of Chicago, Chicago, USA; 4 Population, Family, and Reproductive Health Department, Johns Hopkins Bloomberg School of Public Health, Baltimore, USA; 5 Nigerian Urban Reproductive Health Initiative, Abuja, Nigeria

**Keywords:** Nigeria, Contraception, Risk, Birth spacing

## Abstract

**Background:**

Fertility is high in Nigeria and contraceptive use is low. Little is known about how urban Nigerians perceive the risk of contraceptive use in relation to pregnancy and birth. This study examines and compares the risk perception of family planning methods and pregnancy related scenarios among urban Nigerians.

**Methods:**

A total of 26 focus group discussions with 243 participants were conducted in September and October 2010 in Ibadan and Kaduna. The groups were stratified by sex, age, family planning use, and city. Study participants were asked to identify the risk associated with six different family planning methods and four pregnancy related risks. The data were coded in ATLAS.ti 6 and analyzed using the thematic content analysis approach.

**Results:**

The ten family planning and pregnancy related items ranked as follows from most to least risky: sterilization, abortion, getting pregnant soon after having a baby (no birth spacing), pill, IUD, injectable, having a birth under 18 years of age (teenage motherhood), condom use, having six children, and fertility awareness methods. Risk of family planning methods was often categorized in terms of side effects and complications. Positive perceptions of teenage motherhood and having many children influenced the low ranking of these items.

**Conclusion:**

Inadequate birth spacing was rated as more risky than all contraceptive methods and pregnancy related events except for sterilization and abortion. Some of the participants’ risk perceptions of contraceptives and pregnancy related scenarios does not correspond to actual risk of methods and practices. Instead, the items’ perceived riskiness largely correspond with prevailing social norms. However, there was a high level of understanding of the risks of inadequate birth spacing.

**Trial registration Number:**

This study is not a randomized control trial so the study has not been registered as such.

**Electronic supplementary material:**

The online version of this article (10.1186/s12905-017-0439-2) contains supplementary material, which is available to authorized users.

## Background

In Africa, fertility is historically highly regarded and inextricably linked to women’s value and status [1–3]. Nigeria, with a high total fertility rate of 5.5 and a low modern contraceptive prevalence rate among currently married women of 9.8%, is no exception [4]. Unintended pregnancies in Nigeria are estimated at nearly 1 in 5, half of which end in abortion despite highly restrictive abortion laws [5]. In addition, 16% of married women of reproductive age are estimated to have an unmet need for family planning [4]. Within this context of high desired fertility, highly prized fertility, restrictive abortion laws, and low contraceptive utilization, Nigerian men and women must weigh their desires to control their own reproduction against the risks they perceive to be associated with doing so.

According to a variety of models used to understand health behaviors, perceptions of risk play a key role in driving behavior change [6–9]. Family planning researchers have often employed health behavior models to study contraceptive method use [10–13]. They have paid less attention, however, to one of the key constructs of the models, namely risk perceptions and how such perceptions interplay and compete in reproductive health decision-making.

Within the scope of reproductive health, individuals face a spectrum of competing risks, both real and perceived. These risks exist within a constellation of life decisions that individuals must make in consideration of their own health as well as their family’s wellbeing. Though frequently considered a women’s issue, reproductive health decisions often involve multiple stakeholders, including a woman’s partner, in-laws, or other household members [14, 15], whose motivations may differ from those of the woman herself. Community norms, attitudes, and mores can also influence the reproductive health behaviors of individuals [16, 17]. Depending on one’s individual context, each dimension of reproductive health decision-making (initiation, postponement and spacing of childbearing, family size, family planning use, abortion) can carry additional considerations of risk, forcing women and their families to weigh the known and perceived risks and benefits of each.

In a systematic review of qualitative research focusing on themes that limit contraceptive usage among women, Williamson and colleagues found that perceived risk of sterility was a common theme across five of the seven articles included in their review, and that concerns regarding side effects were universal throughout [18]. In Uganda, young females expressed perceptions of both health-related and more social and cultural risks associated with sexual activity, unwanted pregnancy, unsafe abortion, and early childbearing, while young males expressed risk in terms of bride-price or being “accused of defilement” [19]. In Ethiopia, having a large family was viewed less in terms of the risk it posed to the health of women bearing many children, but rather more so in terms of having less resources available to educate and invest in each individual child [20].

Though recognized as a factor affecting reproductive health decisions, there has been less direct inquiry into how risk perceptions intersect and compete. Men and women may evaluate contraceptives with different priorities, especially in terms of health risks and effectiveness [21]. For some women, the perceived risks they associate with hormonal contraception outweigh the benefits, leaving them unconvinced of the utility of adopting a modern method [22]. For example, in Nigeria, placing oneself at risk of unwanted pregnancy can be looked upon more favorably by some than putting oneself at the risks that are associated with using contraception, both hormonal and barrier methods. Furthermore, in some instances the perceived social consequences of an unwanted pregnancy carried greater weight than any risks associated with abortion [23]. A qualitative study among Malian adolescents found that perceptions of risk played a role in males’ and females’ decisions of whether to use hormonal contraception or condoms [24]. In a review of the literature focusing on adolescent decision making around sexual activity and contraception use, Gage (1998) highlights the ways in which adolescents assess risk of pregnancy and risk of acquiring a sexually transmitted infection, but less so on the perceived risk associated with use of contraception [25].

This study aims to situate family planning within the spectrum of perceived reproductive health risks faced by men and women in Nigeria. By doing so, this information can be used to inform more effective contraceptive counseling and aid in the design of communication programs that can respond to and alleviate the burden that these perceived risks have on the family planning context in Nigeria.

## Methods

### Study design

Qualitative methods, specifically focus group discussions, were used to obtain information on the social attitudes and beliefs regarding family planning as opposed to individual family planning experiences. A semi-structured guide was used to organize and facilitate the focus group discussion. An exercise in the guide was designed to evaluate risk perceptions regarding specific family planning methods and pregnancy-related scenarios. There were six different family planning methods included: condom, pill, injectable, IUD, sterilization, and fertility awareness methods, as well as four pregnancy-related risks: abortion, getting pregnant soon after having a baby (no birth spacing), giving birth under 18 years of age (teenage motherhood), and having six children. Participants were asked to indicate the level of risk each method or scenario poses to health.

For each item, study participants could indicate whether they thought the item was most risky, somewhat risky, or least risky. During the process of categorizing the level of risk for each item the facilitators probed the study participants to explain why they chose the level of risk that they did – and why one item was identified as posing greater or less risk than another (see topic guide in Additional file 1 section). The decision to probe for specific comparisons between scenarios and methods was left to the trained moderators of the focus group discussions, based on the flow of the discussion and the comments shared by the study participants in the moment. The group members were allowed to disagree with each other – and encouraged to engage in discussions around these disagreements. There was no attempt to bring the group members to group consensus about the perceived risk associated with each item.

Although the exercise engaged participants in a discussion about their own perceptions – it was expected that the responses would be influenced by social attitudes and beliefs given that the participants were asked to indicate their choices publicly among their peers as well as asked to voice opinion about methods or scenarios they may or may not have any personal experience with.

### Study population

Study participants included men and women of reproductive age who were residing in Ibadan and Kaduna, Nigeria. The focus group discussions were disaggregated by city, sex, age (18–24 years and 25–49 years), and family planning experience (for women only). The decision to disaggregate focus groups was determined based on secondary analysis of 2008 DHS data from Nigeria, which identified key socio-demographic characteristics influencing fertility and family planning to be north vs. south residence and age (15–24; 25–49). Given laws governing age of consent to participate in research studies, the population was restricted to 18–24 years. The groups were further disaggregated by never-users and current-users of family planning as perceptions and experiences with family planning are likely to differ substantially between these two study populations. A total of 26 focus group discussions with a total of 243 participants were conducted in the two cities in September and October of 2010 (see Table 1).

**Table 1:** Focus Group Discussions by Attribute, Ibadan and Kaduna, Nigeria, 2010

City	Sex	Age	Family Planning Use
Ibadan	Female	18–24 years	Never
			Never
			Never
			Current
			Current
		25–49 years	Never
			Never
			Current
			Current
	Male	18–24 years	na
			na
			na
		25–49 years	na
			na
Kaduna	Female	18–24 years	Never
			Never
			Current
		25–49 years	Never
			Never
			Current
			Current
	Male	18–24 years	na
			na
			na
		25–49 years	na
			na

In order to recruit family planning users into the study, service providers at family planning facilities screened clients for eligibility using a screening form. To recruit never-users, a similar screening form was used at the community level with the assistance of community leaders who mobilized potential study participants. After study recruitment, verbal informed consent was obtained from all study participants before proceeding.

### Procedures

Ethical approval to conduct the study was obtained from the Institutional Review Board at the Johns Hopkins Bloomberg School of Public Health in Baltimore, Maryland, and the Obafemi Awolowo University Ile Ife, Nigeria. Additional approvals were obtained from the state Ministry of Health in the two states where the study was conducted.

### Facilitator training

Master’s level educated, experienced, and qualified research assistants who were native language speakers were recruited and trained to conduct the focus group discussions. The training included the main study objectives and overview of the study, review of the study instruments, techniques in focus group discussion facilitation and note-taking, role of the researchers, recruitment and selection process, field-based data analysis, writing summaries, and research ethics. The research assistants were thoroughly familiarized with the study topic guides through explanation, discussion, and role-plays.

### Data collection

The focus group discussions were facilitated by same sex moderators and note-takers, and conducted in the local language – Hausa in Kaduna and Yoruba in Ibadan. Each focus group discussion was held at place in the community that the study participants identified as conducive to discuss sensitive issues freely. Only the moderator, note-taker, and study participants were present for each focus group. Each focus group discussion lasted approximately 2 h.

### Data analysis

All discussions, with the consent of the participants, were audio taped, and the recordings were transcribed verbatim in the local languages. The transcribed texts were then translated into English. The English version of the transcribed texts were reviewed by the research team – any inconsistencies or issues were noted and communicated with the transcribers. Updated transcripts, where necessary, were reviewed again prior to analysis.

Data sorting, coding, and analysis were carried out using ATLAS.ti software and group level matrices in Excel by one researcher. In addition to using the discussion guide to develop the analytic codes, all transcripts were read to identify emerging themes and allow for the generation of new codes based upon the participants’ own words. Between 2 and 5 codes were utilized for each of the 10 items. A total of 35 codes were used in this analysis. The data analysis, the process of identifying themes, interpreting the findings, and eventually refining the findings, was guided by the thematic content analysis approach [26].

To analyze the results from the risk perception exercise, relative scores for each item (most, somewhat, and least) were tabulated by group discussion – as not all groups covered all 10 items. Next, the scores were weighted by the type of rank and summed into a summary score for each item included. Finally, the summary scores were ranked from most to least risky (see Table 2). The final summary scores were then cross-tabulated by each included demographic factor, and combination of factors, to determine whether the ranking differed by any of those factors.

**Table 2:** Weighted Risk Perception Score Totals by Contraceptive Method and Pregnancy Scenario, Ibadan and Kaduna, Nigeria, 2010

	n	weighted risk score
Sterilization	24	26.63
Abortion	24	26.54
No Spacing	25	24.68
Pill	26	19.62
IUD	24	19.58
Injectable	26	17.73
Teen Mom	24	17.33
Condom	24	17.25
6 Children	24	14.46
FAM	18	1.60

## Results

### Study participants

There were 243 study participants in the 26 focus group discussions. Just over half of the participants lived in Ibadan (54%) and the majority were female (63%). The average age was 27 years. Among the female participants, just over half were family planning users (60%).

### Family planning methods

After ranking the weighted aggregate risk scores from the study participants in regards to the six family planning methods, sterilization was found to be the most risky, followed by the pill, IUD, injectable, condom, and finally, fertility awareness methods (see Table 2). Risk was often categorized in terms of side effects and complications. Sterilization was perceived as much more risky than all other contraceptive methods due to the fact that it requires surgery and is permanent.

*Sterilization is mostly risky because during the process of performing the operation, someone may lose his/her life. It is very bad and dangerous.*

Female, 6 children, family planning nonuser

*It (sterilization) is very risky because it is irreversible. What if an accident happens and the man loses most of his children? I can never advise anybody to do it.*

Female, 3 children, family planning nonuser

In contrast, the use of fertility awareness methods was associated with very minimal risk.

*It (fertility awareness method) is least risky because there is no complication attached.*

Male, 0 children

Pill and IUD risk were considered nearly equal in terms of risk levels – pill use had just a slightly higher risk score than the IUD. Study participants considered IUDs to be superior to pills as the IUD user “doesn’t have to worry about doing things periodically” unlike with pills and injectables.

Some participants noted that the IUD is less risky because it does not contain hormones, in other words, it doesn’t release any chemicals in the body.

*I think IUD is least risky because unlike the pills and injectables it does not leave any chemical substances in the body.*

Male, 8 children

Others characterized the riskiness of IUDs as related to the fact that IUDs can contain hormones – one participant noted the “chemicals” in the IUD can adversely affect the user.

Injectables scored as slightly more risky than condoms; both were found to be much less risky than sterilization, pills, and IUD.

*Most (injectable) users don’t complain of any problems. There is no risk attached.*

Female, 1 child, family planning nonuser

*Injection is good because a woman does not need to take it daily so the risk of forgetting is minimal.*

Male, 0 children

*The condom is better than pills because it has no side effect and is comfortable. I cannot allow my wife to use pills.*

Male, 2 children

### Pregnancy scenarios

The study participants’ risk perception scores resulted in the ranking of the four pregnancy-related conditions from highest risk to lowest risk as follows: abortion, no birth spacing, teenage motherhood, and having six children. Insufficient birth spacing followed closely behind abortion in perceived level of risk.

*Abortion is like a suicide mission…*

Female, 6 children

*It (abortion) is more than dangerous. What if the person dies?*

Male, 3 children

*There is nothing like safe abortion. It can lead to death.*

Female, 20-24 years, 2 children

Lack of adequate birth spacing was seen as risky in terms of the health of the mother and both children.

*[Inadequate birth spacing] is risky because the health of both mother and child are at risk.*

Male, 4 children

Study participants were particularly concerned about the mother’s physical health and the growth and development of the older child – particularly in terms of a shortened breastfeeding interval for the older child.

*It is dangerous because it can lead to the death of the mother and the child. The mother may be very weak and fragile to carry another pregnancy through. The child will be poorly fed.*

Female, 2 children, family planning user

*Very risky because the body is yet to recover from the first pregnancy and now there is another.*

Female, 1 child, family planning non user

*The growth of the child may be stunted and the child may not even enjoy the love and care of the mother.*

Female, 1 child, family planning user

Focus group participants associated much lower risk with teenage motherhood than they did with inadequate birth spacing.

*I have seen someone (of that age) who is already nursing her third child. Children of around 11 years are already rearing children and nothing can happen to them. They will deliver safely with God’s help.*

Male, 3 children

*We have many living examples (of teenage mothers).*

Female, 3 children, family planning nonuser

Having six children was associated with even less risk than teenage motherhood.

*Ah! Many people have more than six children around us and they don’t have any problems.*

Female, 18-24 years, family planning user

The risks noted were mainly in terms of financial ability to feed and provide education for the children. There were a few who mentioned the risk to the mother’s health due to many pregnancies.

*Some parents are rich such that even if they have 20 children they can take care of them comfortably.*

Male, 3 children

### Family planning methods and pregnancy-related scenarios

**Fig. 1:**
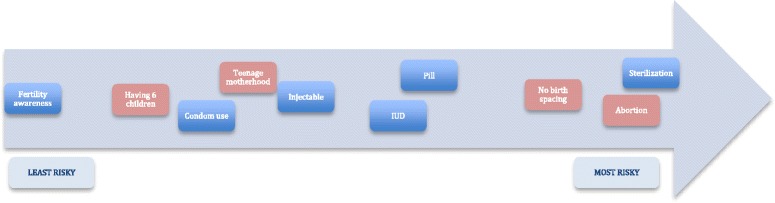
Risk perception ranking of family planning methods and reproductive health scenarios

Considering family planning methods and pregnancy risk items together, participants perceived levels of risk ranked from most risky to least risky as follows: sterilization, abortion, no birth spacing, pill, IUD, injectable, teenage motherhood, condom use, having six children, and fertility awareness methods (see Table 2 and Fig. 1). Sterilization and abortion risk were scored as nearly equally levels of high risk – and no birth spacing followed closely behind in risk level. Giving birth at an age of less than 18 was just slightly less risky than injectable use and slightly more risky than condom use. Although having six children was only more risky than fertility awareness methods – the difference in risk score between the two was quite wide.

When comparing the order of risk of the items by focus group and by characteristics – city sex, age, and contraceptive use – some distinct patterns emerge. Results showed that participants often found sterilization and abortion to be the two most risky scenarios; in fact, the summary risk score by focus group were frequently the same, and the highest, for abortion and sterilization. When the two scores were different, focus groups of women as well as groups with older participants were more likely to perceive abortion as riskiest, while groups of men and younger groups were more likely to perceive sterilization as riskiest. The risk of inadequate birth spacing frequently ranked as the third most risky item – among all Kaduna focus group discussions and more so among the older and female groups in Ibadan than male and younger groups. Using fertility awareness methods was consistently the least risky scenario across all 26 focus groups – having six children was ranked often as the second lowest risk scenario, but not as consistently as fertility awareness methods across all focus groups. Kaduna males often found having six children as being very low risk as compared to the other city and sex groups. Finally, non-users of family planning categorized family planning methods, except condoms and fertility awareness methods, as higher risk than family planning users.

## Discussion

Study participants identified inadequate birth spacing as more risky than all family planning methods, except for sterilization and abortion. This finding gives some insight into the sustained historical high awareness of the risk of short birth intervals among urban Nigerians [27]. Despite this risk awareness, one quarter of births in Nigeria have a birth interval of less than the recommended time of 24 months [4, 28, 29]. Programs that reinforce the risk of inadequate birth spacing as higher than the risk of using family planning might spark an increase in family planning use along with an increase in the average birth interval, if coupled with appropriate support for self-efficacy and service referrals.

Participants ranked both injectables and IUDs as low risk. Injectables were the second most commonly used modern method at 2% in 2008 and the most commonly used modern method in 2013 at 3% [4, 29]. Given the perception that injectables pose low risk to the user in this study and in others, injectable use is likely to continue to gain momentum in Nigeria [30]. IUDs were also perceived as low risk by the study participants. It is possible that the IUD is considered low risk due to the limited understanding of this method, as made clear by variation in comments that surfaced in relation to this method. It is difficult to predict whether a better understanding of this method will lead to a positive or negative change in risk perception. This perception will likely be influenced heavily by how providers present the method to potential contraceptive users [31].

The low risk perceptions of having 6 children and adolescent pregnancy can be tied to perceptions that both are common, and therefore perceived as safe, occurrences as many Nigerians will know of people in these circumstances who are healthy. The identification of these scenarios as lower risk than use of contraceptive methods; however, does not correspond to the actual risk profile. It is much more risky to have a pregnancy in adolescence, and to have many children, than it is to use contraceptive methods for most women. Programs aiming to increase awareness of the risk of having children in adolescence and having many children will need to account for the fact that these are normalized events in Nigeria when designing messages around them.

Because both having many children and using fertility awareness methods are considered low risk and are socially normative, programs can use them as a base upon which to build. Focus can be placed on extending the desire for large families into an appeal for spacing (rather than limiting) births and the acceptance of the practice of family planning using fertility awareness methods into the acceptance of family planning, generally [32].

The difference in risk perception between inadequate birth spacing and having six children warrants further attention and has clear program implications: promoting birth spacing is more likely to be effective in increasing family planning use than promoting limiting births. It is also possible that we can interpret the disparity in the risk perception of these two scenarios as a measure of family aspirations – i.e. that Nigerians desire large, but well-spaced, families. In harmony with the family aspirations could be messaging that highlights avoiding unwanted pregnancies and, therefore, abortion and inadequate birth spacing – two events identified as most risky by the study participants.

It is clear that participants’ perception of risk sometimes does not correspond to actual risk of methods and practices. For example, it is much more risky to have a child as an adolescent or have many pregnancies than it is to use contraceptive methods, for most women. Instead, if grouped into high, medium, and low risk activities, the items correspond with prevailing social norms. The trend suggests that familiarity and the sense that “everyone does it” leads to a low perception of risk. If this is so, increasing the use of contraceptive methods, which are generally seen as riskier than those practices that adhere to socially accepted fertility practices but less risky than engaging in practices that are social taboos, must be informed by people’s perceptions of risk, but not driven by it. Because risk perception is not based on actual medical risk but on other, presumably social norm-related factors, increasing the acceptability of contraceptive methods may be effectively addressed by changing the social norms around contraceptive use in its entirety, rather than by trying to reposition the hierarchy of risks perceived to be associated with individual methods.

Previous qualitative research from five countries, including Nigeria, found that risk associated with contraceptive methods often outweigh individuals’ intentions to prevent a pregnancy that they do not want [33]. Though not explicitly examined in the present analysis, this could suggest an inaccurate calculus placing risks associated with contraception as higher than the risks associated with pregnancy. This is particularly notable given that Nigeria has the 14th highest maternal mortality ratio in the world, estimated at 560 maternal deaths per 100,000 live births, and alone accounts for 14% of all maternal deaths worldwide [34].

This study suffers from a few limitations. The study was qualitative and conducted in just two urban areas in Nigeria – so the findings are not generalizable to the entire urban Nigerian population. Only one researcher coded the data due to time and financial constraints; however, the other researchers assisted in the analysis stage to identify the emerging themes, refine the results, and describe the themes. The risk perception exercise did not include implants, did not specify male or female sterilization, and did not include all types of reproductive health risk scenarios. Additionally, the original study disaggregated the focus group discussions based on more variables than are presented here. Fewer variables were used to disaggregate the groups at the stage of the analysis to compensate for this limitation.

Despite the limitations, there are a few strengths to this study. Though primarily a qualitative study, risk sorting was used in order to quantitatively assess risk perceptions and ranking of risk. Finally, the study analyzed contraception risk perception in comparison to other common reproductive health scenarios – an important factor in health behaviors that is often left out of research.

## Conclusion

Inadequate birth spacing was ranked as riskier than all methods of family planning, except for sterilization and abortion. All modern methods, except condoms, were ranked as riskier than having a baby as an adolescent or having many children. Perceptions of risk did not always follow actual levels of risks, since the pregnancy scenarios discussed are riskier to health than is use of modern contraceptive methods – but more accurately match normative behaviors. Efforts to promote modern methods of family planning will likely achieve most success by focusing on adequate birth spacing – as will efforts to improve the normative appeal of modern methods, generally, as opposed to focusing on specific method attributes.

Understanding how individuals and groups in urban Nigeria perceive risky reproductive health scenarios in comparison to contraceptive methods can help researchers and programmers alike. Researchers can use this information to tailor research questions and tools to better capture prevailing views. This information can also supply programmers with a background to design holistic programs, media materials, campaigns, provider training, and behavior modeling to more widely promote family planning use.

## Additional file

Additional file 1: Topic Guide Demand Gen Draft 2June10. NURHI FGD Generation Demand Topic Guide. This is the topic guide that was used for the FGDs. (DOC 66 kb)

## Abbreviations

NURHI: Nigerian Urban Reproductive Health Initiative
PRHP: Population Reproductive Health Program
