# Supplementary material for: Inadequate birth spacing is perceived as riskier than all family planning methods, except sterilization and abortion, in a qualitative study among urban Nigerians
Source: BMC Womens Health. 2017 Sep 11;17:80. doi: 10.1186/s12905-017-0439-2 (PMC5594467; doi:10.1186/s12905-017-0439-2)
Supplement: Additional file 1: — Topic Guide Demand Gen Draft 2June10. NURHI FGD Generation Demand Topic Guide. This is the topic guide that was used for the FGDs. (DOC 66 kb) [file 12905_2017_439_MOESM1_ESM.doc]

**NURHI Demand Generation Formative Research**

**Topic Guide**

**Draft June 2, 2010**

**Background to the study:**

The Nigerian Urban Reproductive Health Initiative (NURHI) aims to eliminate the supply and demand barriers to contraceptive use in order to increase the contraceptive prevalence rate by 20 percentage points in five years in selected urban areas of Nigeria. The project will be implemented in 6 Nigerian cities. During the first year of the project, several formative research activities are taking place to inform the design and implementation of program interventions. This focus group exercise is one of those research activities.

**Objectives of the study:**

The objectives of this exercise are to:

1. Understand the individual and social barriers to contraceptive use;
2. Explore participants’ perceptions of a happy family and aspirations for themselves and their families;
3. Evaluate demand for integrating family planning services with maternal and child health care, HIV/AIDS services and post-abortion care;
4. Explore the language associated with fertility and family planning to inform message development.

**General guidelines:**

The topics and questions below should be used to guide the focus group. Keep in mind when conducting the focus group to respond to the answers provided by the respondents by asking additional questions or adapting to more appropriate questions.

In particular, there are 3 main approaches to eliciting more information from the respondents:

1. Seek more detail or explanation of a response. For example:
   - Tell me more about ______
   - Can you give an example of _____?
   - What happened next?
2. Explore the reasons behind a response. For example:
   - What makes you say that?
   - What was it about ____that made you decide to_____?
3. Seek clarity and check for inconsistencies. For example:
   - Can you explain what you mean by….?
   - Earlier you said______ but it also seems like______. Can you explain?

Focus Group Discussion Guidelines:

| **INTRODUCTION** |
| --- |
| **Suggested time :** About 15 minutes |

- Thank the participants for coming.
- Explain the purpose of the group discussion:

*We are from the “Nigerian Urban Reproductive Health Initiative” and we plan to be involved with your community over the coming months and years. We’d like to talk with you about families and family planning in your community. We will not ask you about your own behavior, just about your opinions. The information we gather from you and other community members will help us develop and improve the programs we will support in your community.*

- Tell the amount of time the discussion is expected to last – about 2 hours.
- Introduce the facilitator, the note taker and other team members and explain what each one will be doing.
- Explain that a tape recorder will be used since the note taker can’t write down everything.
- Assure that the discussion will be kept confidential. Remind the participants that anything which is said in the discussion should not be talked about outside of the group.
- Explain that there are no right answers and it is okay to disagree. It is important to respect others’ opinions.
- Ask everyone to speak one at a time.
- Read out the consent script.
- Ask if there are any questions.
- Have participants introduce themselves. If they want they can choose a nickname or fictional name to use during the group discussion instead of their real name.

| **ACTIVITY 1 : FAMILY ASPIRATIONS (Technique: Photo elicitation)** |
| --- |
| **Suggested time:** About 40 minutes.  **Materials**: 4 photo cards  ***Note to Facilitator:***  *In this activity, you will use photos of Nigerian families to encourage discussion among group members. To do this, show each photo one-by-one and after each photo, start a discussion using the questions below.*  *The photos are:*   1. *Urban professional middle-class couple, 1 child* 2. *Urban professional middle-class couple, 4-5 children* 3. *Urban couple from poor neighborhood, 4-5 children & extended family members* 4. *Urban couple from poor neighborhood, 2 children* |

Discussion questions/prompts:

1. How would you describe the family in this picture?
2. Do you think they are happy with the number of children they have? Why?
3. What aspirations do you think they have for themselves or their families?
   - probe: e.g. education, health, prosperity
4. Are these aspirations going to be easy or difficult for them to achieve? Why?

| **ACTIVITY 2 : TERMINOLOGY (Technique: Free-listing)** |
| --- |
| **Suggested time:** About 10 minutes.  ***Note to facilitator:***  *In this activity, you will conduct a free-listing exercise by asking participants to think of different words, phrases or idioms that people in their community commonly use to describe the concepts below.* |

Ask participants: What words, phrases or idioms do people commonly use to describe:

1. When a man or woman spaces or limits the number of children they have
2. Birth control you can use to prevent pregnancy up to five days after unprotected sex
3. Taking medicine or having a procedure to end a pregnancy
4. The loss of pregnancy in the first 5 months through natural causes
5. A method or methods used to prevent pregnancy

| **ACTIVITY 3 : DECISION-MAKING FOR FAMILY PLANNING (Technique: Story-telling & discussion)** |
| --- |
| **Suggested time:** About 45 minutes (20-25 minutes for each part)  ***Note to Facilitator:***  *In this activity, you will read out a fictional story about a Nigerian husband and wife.*  *Read out part one, then start a discussion using the questions provided. Next, read out part two, where the story picks up again two months later, and continue the discussion using the questions provided.* |

**Story, part one:**

[Wife name] is 25 years old and married to [husband’s name]. They have 2 girls, the oldest is 4 and the youngest is 1 and a half years. [Wife] has seen a poster at her local clinic about family planning methods that women can use to delay the birth of their next child and thinks it would be good to wait a while before her and her husband have their next child. [Wife] isn’t sure how her husband would feel about this and isn’t sure what to do.

**Discussion prompts:**

1. What are some reasons why the [wife] thinking about using family planning?
2. What do you think will happen next?
3. Who could she talk to about her feelings?

Probes:

- - Why?
  - What could she say?
  - Will this be easy or difficult for her? Why?
  - Would she talk to her husband? Why/why not? What would she say?
  - What would her husband say? Why?
  - In this community, who usually starts the conversation about using family planning?

1. Who has the most influence over deciding when and how many children to have? Why?

Probe:

- - What about the extended family?
  - How involved do you think her husband will be in making the decision to use family planning? Why?
  - How involved should the husband be?

1. If [wife] was your friend, what would you advise her to do? Why?

**Story, part 2:**

Two months later, [Wife] returns to the clinic and talks to the doctor about different methods of family planning. The doctor prescribes her the oral contraceptive pill. She uses this for around 6 months but then stops.

**Discussion prompts:**

1. What do you think prompted [wife] to start using family planning?

Probe:

- - Why motivations do you think she had to start using family planning?
  - Did her husband play a role in the decision to start using family planning? How?

1. Why do you think she stopped using the pill?

Probe:

- - Did her husband play a role in the decision to stop? How?

1. Do you think the oral pill was a good method to use? Why?

Probe:

- - What other methods could she use?
  - Are these better or worse than the oral pill? Why?

| **ACTIVITY 4 : RISK PERCEPTION (Technique: Card-ranking; Discussion)** |
| --- |
| **Suggested time:** About 20 minutes.  **Materials**: 3 risk cards (Most risky, somewhat risky, least risky)  8 action cards  Tape (to tape card to wall) or stones (to hold cards firm on ground)  ***Note to Facilitator:***  *In this activity, you will ask participants to rank a set of actions based on the level of risk they think each action poses to health. Participants will place each action card in order from least risky to most risky. Try to get participants to all agree on the order of the cards. If this is not possible, you can use the majority opinion to make the final decision.*  *This is a good opportunity to energize the group after a lot of discussion so make sure all the participants are involved and moving around to place / move the action cards on the ground or floor.*  *At the end of the activity, ensure the notetaker makes a record of the final ranking.* |

**Step 1:** Place 3 cards on the ground or on the wall – at the far left place “Least risky”, in the middle “somewhat risky”, and at the far right “most risky”.

**Step 2:** One by one, ask participants to place the following picture cards in order from least risky to most risky, guiding discussion for each card using the following prompts. Continue discussion until the group agrees on the order of the cards. Each action should be more risky than the action to its left.

Discussion prompts:

- Why is this action risky?
- Why is ‘x’ more/less risky than ‘y’?
- *For family planning methods (oral, rhythm, condom, sterilization):* Do you think people would be more likely to use family planning if these risks did not exist? What

Action cards:

- Using the oral contraceptive pill to space or limit number of children
- Getting pregnant soon after having a baby
- Getting sterilized to limit the number of children
- Having a birth under 18 years of age
- Using a condom for family planning
- Having 6 children
- Having an abortion
- Using fertility awareness to space or limit number of children

| **CLOSING** |
| --- |

- Thank people for their participation.
- Remind them that the discussion will be kept confidential. Anything said in the discussion should not be talked about outside of the group.
- Provide refreshments
